# The COVID-19 Pandemic and E-Learning: The Digital Divide and Educational Crises in Pakistan’s Universities

**DOI:** 10.1177/00027642231156779

**Published:** 2023-03-17

**Authors:** Sadia Jamil, Glenn Muschert

**Affiliations:** 1 School of International Communications, The University of Nottingham, Ningbo, China; 2 Department of Humanities and Social Sciences, Khalifa University of Science and Technology, Abu Dhabi, United Arab Emirates

**Keywords:** digital divide, COVID-19 pandemic, tertiary-level education, online classes, Pakistani faculty, and students

## Abstract

Recent advances, in information and communication technology (ICT), have significantly impacted some critical sectors of societies (such as transport, health, business, and communication) across many developed and developing countries. Nevertheless, the Internet has proliferated unequally across the world, resulting in global digital inequalities. The onset of the COVID-19 pandemic led to the dependence on online education to prevent the interruption of academic progress in schools and universities worldwide. The global pandemic further worsened the situation for Pakistan, which is neither economically strong nor is the country’s ICT infrastructure well-established to facilitate the successful accomplishment of virtual courses and classes. Thus, this study aimed to analyze the level of Internet access among the Pakistani universities’ teachers and students, and their ICT skills as they applied to online education during the COVID-19 pandemic. The study used the qualitative method of email interviews and thematic analysis to present the study’s results. This study revealed that most Pakistani students, especially those from rural and remote areas, experienced challenges because they neither had proper Internet access, nor could they use laptops and virtual learning systems. Students, who belonged to the upper and middle classes of urban areas and enrolled in private-sector universities, were not as directly affected by Pakistan’s pervasive digital divide to carryout their education during the COVID-19 pandemic.

## Introduction

Information and communication technology (ICT) permeate present-day social relations. Indeed, nearly every aspect of life is affected by and benefits from technology in various ways (Chang et al., 2014). Furthermore, advances in ICT have required people to equip themselves with technology-supported practices to improve their professional and personal lives. However, not everyone in the society has sufficient access to ICTs to do so, a situation which often gives rise to the digital divide problem (Hanafizadeh et al., 2013).

In its earliest iterations, the so-called first-level digital divide referred to the inequalities between groups of people with access to ICT and those without such access (Ragnedda & Muschert, 2013). Scholars argue that this technology-driven gap between haves and have-nots can foster social, economic, and educational inequalities (Hargittai et al., 2019). People with better access to ICT are likely to contribute to society more successfully. Therefore, the digital divide in any society may preclude people from ICT access and effective participation in society. In the past, the digital divide problem limited its scope to physical access to digital technologies such as computers and the Internet. This issue has become problematized because digital life is not just a simple matter of “haves” or “have-nots,” but a vital concern is to investigate aspects other than physical access to the Internet, computers, and smartphones. More recent scholarship has demonstrated a second-level divide as well. In this regard, it is equally important to realize that mere access to ICT may not entail the required skills to engage in a broad range of uses. Thus, digital inequalities manifest themselves at two levels—the first-level divide in terms of access and the second-level divide concerning skills to use the Internet and other ICT devices (Hargittai et al., 2019).

It is more imperative than ever to analyze the digital divide issue regarding ICT access and skills characteristic of individuals from all walks of life, especially in the current circumstances. The arrival of the COVID-19 pandemic has led to the global dependence on online education, a shift intended to prevent the disruption of academic progress in schools and universities worldwide. Hitherto, the effectiveness of online or virtual education has been a contested topic. Some scholars go as far as to contend that online education produces more substantial outcomes than the traditional pedagogical approaches do in physical classrooms (Normore & Lahera, 2019). However, some critics note an inadequate pragmatic base for successful online education, especially in countries where academics and students neither have enough resources to access the Internet and ICT devices at their homes nor skills for practical use (Daniels, 2020; Lau, 2020). The onset of the COVID-19 pandemic has further aggravated the situation for developing countries, which are neither economically strong nor with well-establish ICT infrastructure to facilitate the successful accomplishment of virtual courses and classes.

In Pakistan, all educational institutions were closed for physical classes between mid-March 2020 and May 2020. This past initiative is not surprising, considering the COVID-19 situation and Pakistan’s National Education Policy 2017–2025, which acknowledges virtual education as a way to provide a more significant proportion of Pakistani students with the ICT access and skills required to compete in an information society and to contribute for the economic development of the country. Nevertheless, a digital divide exists across the country. “The country has a total population of approximately 204.60 million and the number of 3G and 4G users in Pakistan has reached to 55.47 million and approximately 150 million Pakistani people use mobile phones” (Jamil, 2022; Jamil & Appiah-Adeji, 2019, p. 43). However, according to the most recent statistics, 78.38 million people use the Internet in Pakistan (Jamil, 2020b; Kemp, 2020). Due to a high level of poverty in the country and infrastructural barriers, high-speed/high-quality Internet services are only available for students in the middle, upper middle, and upper socioeconomic classes living in urban and semi-urban areas (Jamil, 2020a).

Concerning the digital divide in educational settings, past research has mostly paid attention to students’ access to ICTs in schools and homes (Ritzhaupt et al., 2013). Researchers and educators have acknowledged the worth of technology in education, mainly as a means of enhancing teaching-learning processes (Ertmer et al., 2012; Youssef et al., 2013). However, studies analyzing teachers’ and students’ access levels to digital technologies and their skills to use the Internet and ICT devices remain limited in developing countries, such as Pakistan. Previously, different policy documents of education in Pakistan recognize and emphasize ICT use in education, including its potential to support teaching-learning processes (Ministry of Education, 2009). In addition, some studies in Pakistan, offer information on ICT infrastructure at educational institutions and teachers’ views and attitudes about ICT utilization for teaching (Safdar et al., 2011). Nevertheless, no study unpacks the Internet access among Pakistani university teachers and students and their ability to use the Internet and other ICT devices (i.e., skills) to pursue online education during the COVID-19 pandemic. Thus, drawing on the theories of the digital divide, this study aims to fill this gap in the literature.

## Theoretical Framework: The First and Second Levels of the Digital Divide

Today, people experience different sorts of Internet-related difficulties across the globe, ranging from unequal access to the Internet and variations in the skills to use it effectively. Such differences have given rise to undesirable inequalities commonly understood as the digital divide (DiMaggio & Hargittai, 2001; Selwyn, 2004; van Dijk, 2013). Theorists of the first-level digital divide suggest that digital inequalities can be measured by assessing the variance between those who have access to the Internet against those who do not. However, scholars have criticized the binary classification of haves and have-nots. Instead, a new thread of theorizing aims to understand digital inequalities beyond people’s access to the Internet and measuring their digital skills to use ICT devices (Gunkel, 2003; Ragnedda & Muschert, 2013, 2017). Hence, the second-level digital divide focuses on inequalities in digital skills, which indicate a gap between those who effectively use new information and communication tools and those who cannot (Hargittai, 2002; van Deursen & van Dijk, 2008, 2013).

When measuring access to the Internet, a couple of factors are relevant such as reliability of the network connection, affordability, and access location (van Dijk, 2012). Digital skills to deal with ICTs include competencies to operate the Internet and ICT devices, including smartphones, laptops, and computers. Nevertheless, it is crucial to recognize that there are aspects of digital skills beyond rudimentary competencies. Thus, skills vary among people in different contexts and possessing diverse characteristics related to their age, gender, nationality, education, and income levels (Gunkel, 2003, p. 506). Steyaert (2002) offers a notable classification for measuring digital skills: instrumental skills, structural skills, and strategic skills. He suggests instrumental skills as basic skills, which means knowing how to deal with the technology, such as keyboard knowledge (there is a dimension of complexity to these skills). Structural skills refer to the competency with the structure, for instance, the skill to use hypertext (jumping via keywords to other information sources) or looking for dynamic information (via discussion sites, rather than via static information on websites). Structural skills also include using search engines, and especially the capacity to search, find, and evaluate information also fall within this category. Strategic skills include the essential readiness to search proactively for information, make decisions based on available information, and continuously scan the environment for relevant information (Steyaert, 2002, as cited in van Deursen & van Dijk, 2008, pp. 3–4).

The present study uses Steyaert’s (2002) classification to measure digital skills to analyze whether the Pakistani faculty and students have sufficient access and necessary skills to use the Internet and ICT devices (such as smartphones, computers, and laptops) in the pursuit of online education during the COVID-19 pandemic. Such inquiry helps to identify required initiatives to maximize the benefits of ICT in teaching and learning.

## Literature Review

### Understanding the Digital Divide

The digital divide is a multifaceted problem (Chang et al., 2014). It refers to the gap or bridge between the diverse segments of society with easy access to ICT and no or minimal access to modern technologies. With the development of the World Wide Web and multimedia computers, digital inequalities were given much attention in societies across the globe (van Dijk, 2006). This problem prevails in different societies at every level, from the macro to the microscale. Interestingly, it may happen between high- and low-income countries, rural and urban areas, men and women, skilled and unskilled populations, and big and small organizations. This inequality of digital access and skills is intense between developed and developing countries. While digital divide is still widening in most of the world, this is exclusively about the physical access to the Internet and other ICT devices, but it is about having skills to use such access effectively (van Dijk, 2006, p. 2). Concerning this divide, it is essential to understand that digital devices are getting more affordable and easier to use. People with meager incomes are also getting access to these devices, such as smartphones. Nevertheless, the advent of the new and latest technologies and the fact that not all people have access to the latest devices maintain the digital divide problem.

The digital divide issue is not just black and white, and many multifaceted gray areas or factors underpin it. van Dijk (2006) suggests that most inequalities of access to digital resources are not entire inequalities indicating a gap between those who “have” digital access and those who “have-not”; instead, it is of a more relative gap. Some individuals might have complete and easy access, and some might have poor access. Some people might have expert-level skills to use these devices, whereas others might be very novice users with basic ICT skills. Some people might be faster and quicker to adopt the latest technologies, while others might have low adoption levels toward ICT. Thus, in analyzing the digital divide, scholars underline the demographic factors such as age, gender, ethnicity, and socioeconomic status as crucial for the digital inequalities (Ritzhaupt et al., 2013). Some studies reveal that people’s socioeconomic class is also an essential indicator of the digital divide when assessing the individuals’ physical access to ICTs in various studies conducted in educational settings (Hohlfeld et al., 2008).

### An Overview of ICT Development in Pakistan

The past 15 years have witnessed a growth in Pakistan’s ICT infrastructure, as the government has concentrated efforts to develop this area, starting in 2003 (Jamil, 2020c; Yusufzai, 2018). As a result, in terms of Internet users, “Pakistan is the fourth-largest country in Asia—behind Indonesia, India and China” (Hussain, 2017). Nevertheless, many Pakistani people cannot access and use the Internet, and Internet penetration remains worryingly low (Jamil, 2019). The Internet access in Pakistan stands at “around 35 percent, with 78 million broadband and 76 million mobile Internet (3/4G) connections.” According to the Inclusive Internet Index 2019, “Pakistan fell into the last quartile of index countries, ranking 76 out of a 100” (see *The Economist*, 2020). Moreover, the Internet’s range across Pakistan has been far from homogenous. An emerging digital divide (i.e., inequality in the access, use, and impact of ICTs) spans dimensions of gender, income, religion, urbanicity, and education.

### ICT and Its Usage in the Educational Sector of Pakistan

Advances in ICT have become ubiquitous in many developed and developing countries globally, changing how people do their professional work, entertain, socialize, and educate themselves. Notably, the use of ICT has positive consequences for students and teachers, over and above for educational institutions (Lakshmi, 2018; Youssef et al., 2013). The use of the Internet and ICT has become a vital component of education. Scholars widely recognize the significant impact of ICT on teaching-learning processes, content delivery, reaching curriculum goals, and experimenting with digital approaches to education (Amin, 2018; Ertmer et al., 2012). One of the unique aspects of ICT use in the educational field is its use as a collaborative tool that facilitates interactive learning through the Internet and digital devices (such as smartphones and laptops). Specifically, the upsurge of students’ and teachers’ engagement with Web 2.0 has fostered participatory learning processes by sharing knowledge and practicing collaborative writing (Amin, 2018).

Schools can serve as important educational resources in developing the knowledge society (Thunman & Persson, 2013). Specifically, universities and other higher education institutions are often regarded as the robust platforms to produce a skilled workforce necessary to develop an information society. Therefore, the importance of ICT becomes more crucial in universities and other tertiary-level educational institutions to help build a knowledge-based society. However, research on teachers’ and students’ access levels to the Internet and their digital skills needs further development in Pakistan. Thus, this study is unique because it addresses the digital divide concerning online education in Pakistan’s public- and private-sector universities during the COVID-19 pandemic, which has shaken the world economically and socially. Moreover, that has transformed the way people live their lives, interact with each other, and operate their businesses.

## Methodology

### Research Questions and Data Collection

This study used the qualitative method of online interviews in order to investigate two research questions:

• What is the level of Internet access among the Pakistani universities’ teachers and students to pursue online education during the COVID-19 pandemic?
• What are the digital skill levels of Pakistani teachers and students from various universities to use the Internet, ICT devices, and virtual learning systems for online classes amid the COVID-19 pandemic?

Interviewees’ responses arrived via an email questionnaire because of travel restrictions and face-to-face connectivity issues during the COVID-19 pandemic. Researchers used email questionnaires for various reasons. First, extended access to participants was possible through email questionnaires, a mode of asynchronous communication which does not require colocation (Coomber, 1997). According to Bampton and Cowton, “asynchronous communication of time, as is the nature of an interview conducted using email questionnaire, also has obvious advantages as busy interviewees do not have to identify a mutually convenient time to talk to each other” (2002, para. 7). In addition, email questionnaires allowed the interviewees enough time to prepare their responses and an offered them an opportunity to find information that might be significant for the study at hand. While the researchers did not know what resources the interviewees had used to gather information to prepare their responses, email questionnaire was found a significant method to collect data during the COVID-19 pandemic (See also Bampton & Cowton, 2002, para. 8).

Notwithstanding these advantages of carrying out interviews using email questionnaires, there were some disadvantages that both researchers acknowledged. For example, an email questionnaire “provides a limited register for communication,” which means it does not allow the researcher to observe the cues and facial expressions of the interviewee that can unpack many realities of the issue under investigation (see Bampton & Cowton, 2002, para. 25). Also, as aforementioned, interviewees could use diverse known and unknown sources to construct their responses for the email questionnaire, which might affect the quality of original data. However, given that this study occurred during a challenging global pandemic scenario, the data were gathered via the best available methodology for contacting the study’s participants.

### Sampling of Research Participants

Using snowball sampling, the researchers conducted 23 interviews of faculty teaching staff members (seven females and sixteen males) from Pakistan’s public- and private-sector universities. The selected interviewees are of age ranging between 28 and 61 years. The purpose of snowball sampling was to develop contacts with research participants and informants, who could help the researchers to contact faculty teachers and students for data collection (see also Fortune et al., 2012).

### Sampling of Universities in Pakistan and Their Regulatory Mechanism

Using snowball sampling, the researchers chose some Pakistani universities to contact research participants. Table below, lists the interviewees’ universities in this study.

**Table.** Sampling of Interviewees in This Study.

	Sampled universities
1.	Bahria University
2.	University of Management and Technology, Lahore
3.	University of Karachi
4.	Punjab University
5.	Peshawar University
6.	National University of Science and Technology
7.	Balochistan University
8.	Hamdard University, Karachi
9.	Sindh University of Jamshoro
10.	Iqra University, Karachi
11.	COMSATS, Lahore and Islamabad
12.	NED University, Karachi

As far as the functioning of Pakistani universities is concerned, all higher education institutions operate as semiautonomous bodies commissioned by federal and provincial governments. All higher education institutions in the country operate under the policy guidelines set by the Higher Education Commission (HEC), established in 2002, replacing the University Grants Commission through an ordinance issued by the President of Pakistan (Government of Pakistan, 2002). The purpose of this legislation has been to drive the promotion of higher education, research, and development in the country. HEC is responsible for formulating and facilitating policies, plans, guiding principles, programs, priorities, and higher education standards leading to the country’s socioeconomic development (Ministry of Education, 2009). Public-sector institutions mainly contribute to the higher education of Pakistan. There are more than 160 public- and private-sector HEC’s recognized universities across the country.

### Data Collection Procedure and Limitations of the Study

The research participants responded to the questionnaire in April 2020. Initially, 43 faculty teaching staff members in Pakistan’s different public and private universities received invitations to participate via phone calls and emails. Unfortunately, many potential interviewees could not participate because of their challenging personal circumstances due to the COVID-19 pandemic and the nationwide lockdown. Those, who agreed to the interview, were mainly comfortable in responding to the email questionnaire. However, it was difficult for many of them to manage Zoom or Skype interviews due to their strict schedules and unpredictable power failures.

Each interviewee responded in English language. First, respondents described their own and their students’ experiences related to access to the Internet within and outside the workplace/university (i.e., research question one). Second, subjects described their digital skills for the use of ICT devices (i.e., smartphones, laptops, and desktop computers), online learning platforms (e.g., Moodle and Blackboard), and meeting platforms (e.g., Zoom and Microsoft Teams) (i.e., research question two).

### Data Analysis: Inductive and Deductive Thematic Analyses

This study used thematic methods to analyze the gathered data under two key themes that emerged inductively from the interview data, namely:

— Teachers’ and students’ access to the Internet for online education; and
— Teachers’ and students’ digital skills to use the Internet and other digital tools.

The sub-themes emerged via a deductive thematic analysis, which refers to using a predetermined framework (Flick, 2009). In this study, the thematic analysis employed Steyaert’s (2002) conceptual framework to classify teachers’ and students’ digital skills across three deductive subthemes: instrumental, structural, and strategic skills.

### Research Ethics

To preserve research ethics, all interviewees received a project information sheet that provides information about the objectives of this study, methodology, types of research questions with examples, voluntary participation, the confidentiality of their names and institutions, and access to research findings. To preserve privacy, any discussion refered to interviewees using pseudonyms.

## Findings and Discussion

### Teachers’ and Students’ Access to the Internet for Online Education During the COVID-19 Pandemic

The global onset of the COVID-19 pandemic and subsequent lockdowns compelled many colleges, schools, and universities to cancel in-person classes and begin teaching their courses online. In the South Asia, universities in India, Bangladesh, Pakistan, Sri Lanka, and Afghanistan, found themselves not fully prepared for delivering online education. In many cases, students from rural areas were without the Internet or proper facilities for online education. On March 18, 2020, higher education authorities in Pakistan advised all universities to organize online teaching and learning, as educational institutions were closed down for in-person classes (Khattak, 2020). This was not the first time when universities had experienced the closure. In the past 15 years, Pakistan witnessed university closures several times because of terrorism attacks and volatile political situations. Nevertheless, universities had never adopted online teaching before. An unexpected shift to digital learning amid the coronavirus pandemic posed some challenges to the country’s educational system. Most students did not have computers or laptops, nor did they have access to the Internet facilities such as Web cafes. This study’s feedback, from instructors, revealed that most Pakistani students did not have access to ICT devices and the Internet for online classes. Some students relied substantially on smartphones to access the Internet, especially in remote and rural areas. However, impediments included low Internet speed, high cost of Internet service, and poor mobile signals. In this regard, a male faculty staff member from a public-sector university of Khyber Pakhtunkhwa province suggested:

About 50 percent students don’t have access to the Internet. On the other hand, students who can access to online classes, are mostly using 2G Internet and their download speed is below 2mbps. Besides, a majority of students, who have the Internet connectivity, are using smartphones. Over 60 percent of our students belong to remote areas of district Swat, district Shangla and district Buner, where the Internet connectivity is a big issue. Despite of the major cities, other hilly areas of above-mentioned districts lack the Internet facility, and students residing there are depended on cellular phone services, which have poor signals. (Interviewee ABC)

This study noted that thousands of students across Pakistan’s Khyber Pakhtunkhwa province, Gilgit Baltistan, Federally Administered Tribal Areas (FATA), Azad Jammu, and Kashmir were unable to access the Internet for online classes at home. They had to travel miles every day amid the pandemic to take online classes in government-designated buildings, which was not safe for them. Students, who lived in these areas, were deprived of Internet connectivity in the absence of 3G and 4G and looked forward to the Pakistani government providing improved Internet services. During the pandemic, a Pakistani court had instructed the government to provide Internet services to northwestern Khyber Pakhtunkhwa Province, where a lack of Internet service deprived thousands of university students of access to online classes and educational content (Gandhara, 2020). When talking about the situation of online education in different provinces of Pakistan, interviewee JKL from the University of Karachi highlighted:

ICT infrastructure, in Pakistan’s major cities and in all provinces, has considerably improved over the years. The quality of ICT infrastructure and the speed of Internet may vary between provinces, regardless the Internet service provider. Punjab and Sindh provinces are much better in terms of ICT infrastructure, the speed and the reliability of Internet connection. Students and teachers, in Balochistan and Khyber Pakhtunkhwa provinces, are more in problem ever since COVID-19 has disrupted on-campus teaching. Also the level of students’ and teachers’ affordability of the Internet cost may vary. Affording a costly Internet connection may not be a problem for teachers and students at private-sector universities. But trust me, instructors are not well-paid at public-sector universities across Pakistan. Then mostly students, belonging to middle or lower middle class, study in public-sector universities. It is hard for them to afford expensive mobile Internet packages.

Internet affordability is one of the most significant barriers in Pakistan. One economically viable option for students and teachers is to use broadband connections, which are comparatively cheaper than mobile Internet packages. Nevertheless, an interviewee from the Institute of Business Administration suggested, “broadband services are not quite reliable in Pakistan. I use broadband services of Pakistan Telecommunication Limited. No one attends in their offices, in case of any service interruption” (Interviewee OPQ).

This study further noted that the Pakistani universities and government do not support students in pursuing online education. Even students, who belonged to the middle and upper middle classes in urban areas and who could afford the Internet service, had also faced challenges accessing the Internet during the COVID-19 pandemic. For example, according to a male faculty staff member belonging to a private university in Punjab province:

We have some examples that universities are conducting online semester and exams during the pandemic, such as Dow Medical College (Karachi), Institute of Business Administration (IBA—Karachi), Bharia University (Karachi, Lahore and Islamabad), and some other higher education institutions. However, a majority of students don’t have the facility of landline/broadband Internet connections. Some of them use mobile Internet, which does not allow the customer to download freely and it also consumes a lot of Internet data while taking online classes. Most of the students are seen taking their classes on their smartphones. Many students send text messages to their class representatives to request the teachers for giving them exemption to take online classes as they are facing Internet problem. It requires a strong Internet connection to join online class and the applications/soft wares consume heavy data. I don’t face much difficulties while conducting online classes, but I always face the problem from students’ side regarding poor Internet and a lack of basic points of access. Many of the students reside in villages or in small towns, where 4G Internet has not been provided by the cellular companies. I have to wait for a long time to start my lecture because students join often late. (Interviewee DGL)

Contrary to the views of Interviewee DGL, a female faculty teaching staff member from a private university underlined:

In private sector universities, students have way better facilities than public sector universities. They have many gadgets with them and they can afford these all facilities as they afford studying in private sector universities, which already provide students with all the facilities as students are their customers and they are meant to serve them for customer care. But this is not the case in public sector universities because these are government’s owned institutions. Their fee structure is very low. A majority of students, who study in public sector universities, belong to low class background. They do not have luxuries and gadgets, and they rarely get Internet access. It is really challenging to gather them at a platform for online class. (Interviewee FDG)

Indeed, the finding supported the notion that, due to better economic resources, Pakistani students who studied in private-sector universities could better access the Internet and ICT devices. Nevertheless, digital connectivity is not solely about access to the Internet, and it has some other essential facets. A vital dimension of Internet access is the reliability and the quality of the connection (Khan et al., 2020; Selwyn, 2004), which is undoubtedly absent in most areas in Pakistan.

Furthermore, respondents’ feedback suggested that faculty teaching staff members could enjoy comparatively better access to the ICT devices because they could afford to use personal laptops and smartphones to access the Internet and teach online. However, they still experienced the Internet connectivity issues. For example, a faculty teaching staff member from a public-sector university in Sindh mentioned that “I am using 3G Internet service and laptop for my online sessions at home. Sometimes due to low Internet speed and connectivity problem it is very difficult to upload online video lectures and data” (Interviewee RKJ). Similarly, another female respondent from a Sindh-based university highlighted, “Internet access is the biggest problem in Pakistan. I have broadband connection of Pakistan Telecommunication Limited with 4MB speed, but still I face the problem of frequent disconnections at regular intervals” (Interviewee LMN).

Pakistan is already experiencing the first level of the digital divide, and many Pakistani people do not have access to the Internet for several reasons (Jamil, 2020a). This study found a very relevant power failure problem, which affected the teachers’ and students’ access to the Internet in Pakistan. According to a female respondent from a public-sector university in Punjab province, “technological problems are another challenge faced during an online class. Due to electricity load shedding and uncertain Internet conditions in Pakistan, it is very difficult to ensure that every student and teacher has an access to technology” (Interviewee HOP). Notwithstanding issues of Internet connectivity and students’ lack of access to ICT devices, this study did not find any supportive role of universities and the HEC of Pakistan to resolve this problem. “In this pandemic situation, neither HEC nor universities have supported us to ensure the Internet access. Students and teachers are facing difficulty in terms of Internet access,” underlined a faculty member at a public-sector university in Punjab province (Interviewee SOW).

This study also found that online education and a shift to work from home across the country had swiftly increased the burden on the Internet network in Pakistan. Consequently, Internet speeds were slowed down in many parts of the country. The slowdown compounded frustration with low-quality online learning, and caused mass frustration among students and teachers. In the COVID-19 pandemic, this study further revealed that access to the Internet was not just a gap between haves or have-nots. Instead, it was a relative sort of gap that depends on students’ and teachers’ access point location, economic resources, type of educational institution, and the Internet service provider for reliability or quality of the Internet. In addition, this study found a crucial political aspect to issues of access, particularly in the case of Pakistan. Specifically, some respondents mentioned about the government’s restrains on the Internet access in the northern province of Khyber Pakhtunkhwa, FATA and some other parts of the country. During the pandemic, such restrictions caused thousands of students and teachers to suffer from educational crises.

Considering that online education was experimented with for the first time in the history of Pakistan, this study invoked to look at whether university teachers and students can be better prepared to teach and learn online, respectively. Therefore, the following section addresses teachers’ and students’ skills to use the Internet, ICT devices, and software to facilitate online classes in the country.

### Teachers’ and Students’ Digital Skills to Use the Internet, ICT Devices, and VLS for Online Education During the COVID-19 Pandemic

It is no wonder that the use of the Internet and technology is now crucial to daily life, so it is also no surprise that digital skills are essential for everyone. Digital skills of various types may seem simple, but skills deficits pose many issues to teachers and students. Indeed, digital skills are fundamental in the education sector, currently under digital transformation in many parts of the world (Starkey, 2012; White, 2015). The arrival of the coronavirus pandemic principally altered the way teachers and students carried on their education process, which was no more limited to face-to-face or in-person classes. Now the world is witnessing virtual learning systems (VLS) as the vital source of education across the globe. Such facility requires teachers and students to be well-equipped with the necessary digital skills and their comfort level to use the technology. Still, even if students and teachers are comfortable with technology, teaching and learning online necessitates its procedures, many of which can be new to teachers and students used to in-person teaching and learning in classrooms. More than ever, teachers and students need digital skills to use the Internet, ICT devices, and VLS effectively to compete with the new pedagogical demands. Unfortunately, in many developing countries (such as Pakistan), teachers and students are ill-prepared for online teaching and learning due to a lack of digital skills (Khattak, 2020).

In Pakistan, the prevalence of the second-level digital divide in different sectors such as education, business, health, and economics is ubiquitous (Jamil, 2022; Yusufzai, 2018). Notably, the onset of the COVID-19 pandemic had triggered this debate because the country’s higher education system was suffering from severe crises. Based on Steyaert’s classification of digital skills (2002), this study revealed that students, who were living in remote or rural areas of Pakistan, substantially lacked instrumental and structural skills to operate the Internet, laptops, VLS (such as Moodle and Blackboard), and online meeting portals (such as Zoom). However, they could operate smartphones well and they did receive educational material through WhatsApp and email, which indicated that they were deprived of facilities, but not wholly excluded. For instance, an interviewee stated:

Students, in rural areas and remote areas of Federally Administered Tribal Areas (FATA) and Khyber Pakhtunkhwa have just an option of smartphone to receive learning content. Though they are neither skilled enough to use laptops, Moodle or Blackboard, and nor most universities here use these Virtual Learning Systems (Interviewee TSP).

Students, who were residing in urban areas, possesed the necessary instrumental and structural skills to operate the Internet and browse and evaluate learning materials. For example, an interviewee from a public-sector university in Sindh province highlighted that “the students, living in main city areas, have acquired better digital skills than those living in remote areas” (Interviewee CDB). Moreover, this study demonstrated that Pakistani students lacked strategic skills to benefit from using the Internet for online learning. Describing the level of digital skills among the Pakistani students, an interviewee from a public-sector university in Punjab province said:

Mostly students do not have virtual learning applications or programs installed in their gadgets and many do not understand its usage, which at time become really challenging. It is a new experience for everyone especially students and they do not know how to effectively use technology for learning purpose. (Interviewee ULM)

A worrying matter was the lack of digital skills among the faculty teaching staff who did not digital competencies necessary to carrying out online classes. “Do not know about virtual learning system, but I am digitally skilled,” says a faculty teaching staff member from a public-sector university in Khyber Pakhtunkhwa province. When commenting about the digital skills of instructors, a female faculty of a private-sector university in Karachi revealed:

We waited one semester to see whether to give training to the staff because campus was closed in April 2020. It is difficult because not everybody is skilled enough to use Blackboard. We did have training sessions for instructors in Fall Semester of 2019. In some other private universities, management have arranged sessions for streamlining possible options for online teaching. Nevertheless, the situation in public-sector universities is worrisome because they do not have well-trained staff even to operate through Zoom. (Interviewee TRP)

Likewise, a male interviewee from a public-sector university expressed his concern over the digital skills of instructors and students. He suggested the following:

I work at a public-sector university of Punjab province. I had never used Zoom ever in life until my colleagues suggested me to try it in order to continue teaching online. We did group sessions at our home to learn how to operate Zoom for conducting online classes. My University is the largest public-sector university of Pakistan’s Punjab province and we do not use Virtual Learning Systems until so far. Therefore, there are no tutorials for instructors and students for online education. Another challenging step was to see whether all students could attend Zoom classes. Trust me, only 40 percent of my students could use Zoom for online classes during Spring Semester, 2020. Rest had to rely on group teaching (face-to-face), which was risky due to pandemic. Now the situation is much better. Most teachers are relying on Zoom for their classes and slowly they are getting skilled for online teaching. But still we cannot compete with international universities in terms of instructors’ training. (Interviewee WEX)

These findings suggested that faculty teachers might have basic instrumental skills to operate the Internet and ICT devices; however, they might were lacking advanced skills to use VLS and online meeting portals. Unfortunately, the apex educational body (i.e., HEC) had lapses in ensuring Internet access in many parts of the country. It had not firmly instructed public- and private-sector universities to provide their teacher training to conduct online classes. Respondents’ feedback unpacked that many faculty members had to rely upon their resources to facilitate online classes. For instance, an interviewee from a private-sector university in Punjab province stated:

I have received my Master degree from a well-reputed foreign university, where online classes used to be a routine. Being a teacher, it is a new experience to teach online in Pakistan, but I have good skills of delivering online classes. Initially, I used a trial version of ZOOM, which did not provide unlimited/required time meeting duration. So, lecture automatically discontinued after 40 minutes. It took me too much time to be in the learning process again and to conduct online classes. I am doing all these efforts myself. A lack of a good Internet connection has always been a challenge too ever since we have started online classes. (Interviewee WOP)

Digital tools and online educational programs are constantly changing the way teachers teach and students learn. In Pakistan, this study demonstrated that teachers in higher education lacked strategic skills that were crucial to keep up with evolving technology, to know what digital tools are best suited to their students, and to use them effectively in their online teaching. Virtual reality classrooms, for instance, are a state-of-the-art tool that can permit students to experience the entire world from within the confines of the classroom. However, unfortunately, the interviewed Pakistani faculty teachers were neither digitally skilled nor they were prepared to accept this transition.

Noticeably, this study highlighted that male academics were more motivated to develop their instrumental, structural, and strategic skills. Female academics, in comparison, were both professionally and personally constrained due to their shouldering a majority of domestic responsibilities. As a result, interviewed women academics had less time available to develop skills. Their male counterparts might not grasp this relative barrier experienced by women instructors in Pakistan, as one male respondent from Punjab province clarified, “It is a tough time, especially for female teachers who have domestic responsibilities as well. However, I believe we can develop many skills using our personal resources and can facilitate students for online classes” (Interviewee YOP). In contrast, the female instructors were well aware that they might lag in instrumental and strategic skills, just as they cited the strained work-life balance as its cause. Concerning this issue, a female faculty staff member from a public-sector university in Sindh province mentioned:

Women have to struggle at different ends. We have to take care of family, and then we have to struggle for opportunities too. We must tell our society that men and women both need to be equally digitally skilled for the bright future of Pakistan. (Interviewee FMN)

The point about the lack of opportunity indicated problems for female Pakistani academics not only within their professional academic lives but also at the societal level, where offering more opportunities to men is a generalized stance.

## Conclusion

This study addressed the educational crises faced by Pakistan’s public- and private-sector universities to facilitate online classes during the COVID-19 pandemic. The country’s pervasive first-level and second-level digital divides affected the teachers’ and students’ capacity to continue online education when in-person classes were not possible due to the pandemic lockdown. This study revealed that the Pakistani students, especially those from villages and remote areas, were the recipients of inferior online education, without proper Internet access and ICT devices, and without skills effectively to use laptops and VLS. Students, who were belonging to the upper and middle classes of urban areas and who were studying in private-sector universities, had not experienced directly the educational crises caused by Pakistan’s pervasive digital inequalities. However, they did experience some common problems, including the poor quality of Internet service, access point issues, and a lack of opportunities to have digital skills training for both male and female students. As far as the teaching faculty was concerned, they had comparatively better access to the Internet and ICT devices than their students during the pandemic. Nevertheless, some teachers lacked sufficient skills to conduct online classes using advanced VLS.

The Pakistani government should prioritize substantial investments to bridge the digital divide and to meet the technical needs of higher education institutions and students. Such an investment in infrastructure and skills development in the digital sphere could combat the impact of the COVID-19 pandemic on Pakistan’s public- and private-educational sector. What is worrying is a lack of robust public discourse on guaranteeing constant educational provision to students amid the pandemic-induced shutdown of universities. Also, investments by the government and initiatives by HEC are unlikely because the ruling authorities’ current focus is on safeguarding the basic survival needs of the country’s impoverished people, who rely on daily wage income in the informal economy.

Pakistan is currently confronting an unprecedented situation that threatens to undo hard-won gains in education from the past decades. Although not unique in its challenges, the country has a weak weak economic structure. It does not currently have the infrastructural or financial capacity to immediately establish or sustain virtual learning, especially for teachers and students in remote and rural areas. To effectively overcome the crisis, education professionals at all levels must optimize the effectiveness of available resources.

In conclusion, it is very important to mobilize the resources so as to develop and deliver ICT infrastructure and equipment in Pakistan. The government and HEC need to develop and deploy guidelines and best practices for how teachers and students can pursue online classes and surmount the existing digital divide. Such guidelines should consider both first-level and second-level challenges to online education posed by the digital divide as it exists on the ground. It is insufficient to keep pace with emerging virtual teaching and learning standards in other countries, as external digital challenges may be incomparable to those in Pakistan. Thus, it is crucial to develop country-specific (or even region-specific) initiatives considering available resources. Pakistan must deploy strategies to improve digital skills of all types, including instrumental skills (i.e., to operate the Internet, ICT devices, and virtual learning programs), structural skills (i.e., to handle the digital tools and devices), and strategic skills (i.e., to benefit from the usage of the Internet for online classes).
